# Telehealth multicomponent exercise and health education in breast cancer patients undergoing primary treatment: rationale and methodological protocol for a randomized clinical trial (ABRACE: Telehealth)

**DOI:** 10.1186/s13063-022-07015-z

**Published:** 2023-01-19

**Authors:** João S. Henkin, Cíntia E. Botton, Mariana S. Simon, Guilherme G. Rocha, Caroline B. Silveira, Ricardo S. Gehrke, Gabriella B. Freitas, Gabriel S. Trajano, Ronei S. Pinto, Stephanie S. Pinto

**Affiliations:** 1 Exercise Research Laboratory (LAPEX), Physical Education, Physiotherapy, and Dance School, Universidade Federal do Rio Grande do Sul (UFRGS), Rua Felizardo, 750 – Bairro Jardim Botânico, Porto Alegre, Rio Grande do Sul CEP: 90690-200 Brazil; 2 Exercise Pathophysiology Research Laboratory, Hospital de Clínicas de Porto Alegre, Clinical Research Center, Porto Alegre, Rio Grande do Sul Brazil; 3 School of Exercise and Nutrition Sciences, Faculty of Health, Queensland University of Technology, Brisbane, Australia; 4 Neuromuscular Assessment Laboratory, Physical Education School, Universidade Federal de Pelotas, Pelotas, Rio Grande do Sul Brazil

**Keywords:** Breast neoplasms, Physical exercise, Resistance training, Telehealth, Telemedicine, Telerehabilitation, Mobile health, mHealth, Digital intervention, Fatigue

## Abstract

**Background:**

Current guidelines emphasize cancer patients should increase their physical activity levels, encouraging physical exercise practice as a complementary therapy to mitigate adverse effects during treatment. Telehealth can be a feasible method to improve adherence and interventional support for breast cancer patients, of which most do not meet sufficient physical activity levels after diagnosis. The Adaptations to Breast Cancer and Exercise Using Telehealth (ABRACE: Telehealth) study aims to investigate the effects of a 12-week telehealth multicomponent training program plus a health education program (MTHE), compared to a health education program alone (HE), on physical and psychological outcomes in breast cancer patients undergoing treatment.

**Methods:**

This study is a randomized controlled trial. Women undergoing primary treatment (during or after chemotherapy) for breast cancer (stages I–III) will be randomly assigned to MTHE (twice a week) or HE (once a week). MTHE components are mobility, aerobic, balance, resistance, and flexibility home-based exercises, supervised by video call. The primary study outcome is cancer-related fatigue. The secondary outcomes are quality of life, symptoms of depression and anxiety, physical activity level, cancer-related cognitive impairment, and functional capacity. Other outcomes are adherence to interventions and a follow-up questionnaire evaluating the individual perception in motivation, lifestyle changes, and main barriers to participation. All outcomes will be remotely assessed before and after intervention. Our analysis will follow the intention-to-treat approach and per-protocol criteria, with additional sub-group analysis.

**Discussion:**

To our knowledge, this is the first randomized clinical trial in breast cancer patients using a face-to-face videoconference strategy to supervise physical exercise. Our hypothesis is of superiority for the effects of MTHE on primary and secondary outcomes compared to the effects of only the health education intervention.

**Trial registration:**

Adaptations to Breast Cancer and Exercise Using Telehealth (ABRACE: Telehealth), NCT04641377. Registered on 23 November 2021, https://clinicaltrials.gov/ct2/show/NCT04641377

**Supplementary Information:**

The online version contains supplementary material available at 10.1186/s13063-022-07015-z.

## Background

Cancer is the second leading cause of death worldwide, with breast cancer being the most frequently diagnosed type [50, 57]. Although breast cancer incidence rates are 88% higher in developed countries, the mortality rates are 17% higher in developing countries compared to developed countries [50]. The advancements in early detection methods and more effective treatments have contributed to a higher survival rate in 5 years and a decline in mortality in this population in developed countries [2, 12].

Chemotherapy as part of primary cancer treatment increases the chances of survival [21]. In contrast, there is a need to counteract health issues related to disease and toxicity of treatments, which can lead to short- and long-term side effects in cardiovascular, neuromuscular, and immune systems [27, 30, 53]. Additionally, the cancer treatment can increase cancer-related fatigue, impair quality of life, and negatively affect mental health and patients’ ability to perform daily activities [4, 25, 27].

Physical exercise has been shown as a complementary therapy during breast cancer treatment aiming to mitigate the adverse effects of medication and improve patients’ quality of life [9]. Current exercise guideline for cancer survivors [9] recommends moderate-intensity aerobic training at least 3 times per week, for at least 30 min, in addition to twice-weekly resistance exercise comprising one exercise per major muscle group, 8 to 15 repetitions per set, 2 sets per exercise. Moreover, patients should avoid inactivity and increase physical activity progressively according to their ability and health condition [22]. Despite these recommendations encouraging an active lifestyle, only 11–19% of cancer patients meet sufficient physical activity levels after the diagnosis [6, 52]. Some perceived barriers include the adverse effects of treatment (e.g., fatigue), low motivation, lack of time, lack of access to fitness facilities, and low social support [14]. Also, most oncology patients prefer exercising at home [14, 59]. In addition, training supervision is an advantageous strategy to improve health outcomes, such as quality of life, physical function, anxiety, and depression symptoms, compared with non-supervised exercise in cancer patients [7, 9, 51].

As an alternative methodology, telehealth is an important tool to improve the monitoring and delivering of a physical exercise program for cancer patients, besides being safe and low-cost [5, 48]. Telehealth can be defined as distance-based interventions delivered using information and communication technologies to assess, educate, monitor, and/or guide physical exercises or other health care interventions [5, 45]. However, the efficacy of home-based exercise on fatigue and psychosocial outcomes in breast cancer patients undergoing primary treatment is not entirely established. A meta-analysis including seven unsupervised home-based interventions [55] found no positive effect on cancer-related fatigue in patients ongoing breast cancer treatment. On the other side, a recent meta-analysis study including cancer survivors demonstrated that home-based physical activity interventions induced small improvements in fatigue for up to 9 months [23]. It is important to highlight that the participation rate in these home-based interventions was higher (mean 55.33%) than in previous studies during (41–43% )[11, 54] and after (37%) [26] treatment. Although adherence to exercise is a positive feature in home-based interventions, the heterogeneity among exercise protocols is remarkable regardless of the environment [59]. Previous interventions did not attend principles of training and exercise recommendations as an indication of the quantity and multiples components (i.e., mobility, aerobic, balance, resistance, and flexibility exercises). Also, these studies failed to adequately report exercise prescription variables (i.e., frequency, intensity, time, and type), hindering the protocol application by health professionals and the comparison between studies [15, 39].

Therefore, our study aims to evaluate the effect of telehealth multicomponent training plus a health education program, compared to a health education only, on fatigue, quality of life, anxiety and depression symptoms, and functional capacity in women undergoing primary treatment for breast cancer. The choice of the active comparator group intends to reach the principle of equipoise and partially accounts for research participation effects (Hawthorne effect). Our central hypothesis is of superiority for the effects of multicomponent training plus a health education program group (MTHE) on primary and secondary outcomes compared to the effects of only a health education program (HE).

## Methods

### Protocol registration

**Fig. 1:**
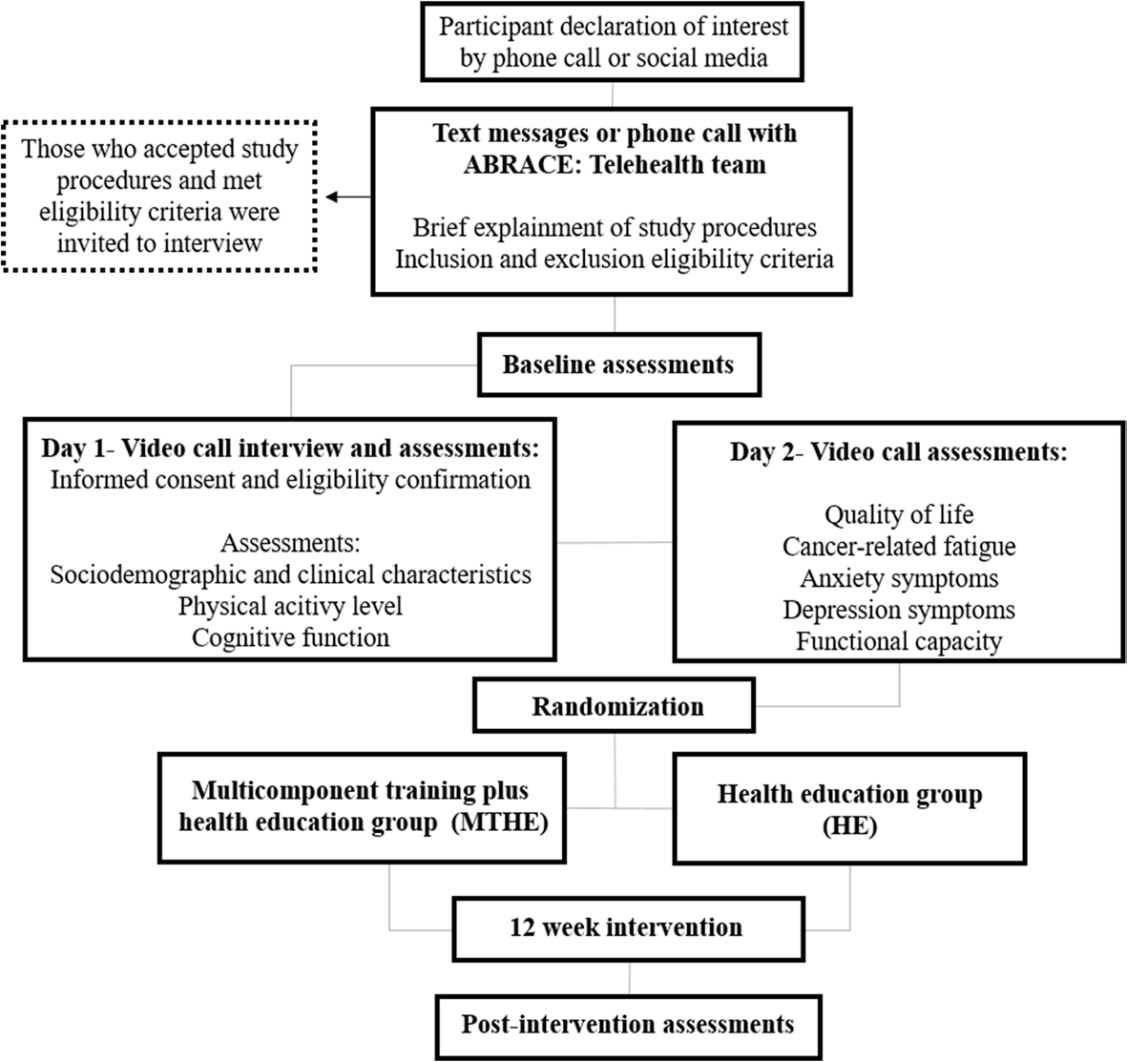
The ABRACE: Telehealth trial design

The *A*daptations to *Br*e*a*st *C*ancer and *E*xercise Using Telehealth (ABRACE: Telehealth) study is a two-armed randomized controlled trial registered on Clinical Trials (NCT04641377) on November 23, 2020, before the first participant enrolment (i.e., on February 26, 2021). The planned flow diagram of this trial is presented in Fig. 1.

### Study setting and eligibility criteria

This trial will take place in Porto Alegre and Pelotas, cities located in southern Brazil. The research team and the necessary training equipment present in the interventions are from *Universidade Federal do Rio Grande do Sul* and *Universidade Federal de Pelotas*. The protocol study will be conducted following the SPIRIT (Standard Protocol Items: Recommendations for Interventional Trials) statement [10]. Participants will be recruited through advertising on social media, in local newspapers, and at hospitals and oncology clinics in the cities involved in the study. After initial contact with researchers, volunteer will schedule an interview with the one of the researchers. The participants will be inquired about all eligibility criteria by a trained evaluator through a video call, which will be performed individually to ensure the eligibility of the participants. Inclusion criteria for subjects are defined as follows: (1) women living in Porto Alegre or Pelotas; (2) aged 18 years or older; (3) medical diagnosis of breast cancer in stages I-III [3]; (4) volunteers undergoing primary treatment for breast cancer (i.e., adjuvant or neoadjuvant chemotherapy; or radiotherapy or immunotherapy after chemotherapy); (5) physically inactive at least three months prior to eligibility (i.e., women who do not reach current physical activity guidelines of at least 150 min of aerobic and/or muscle-strengthening activities on 2 or more days a week) [8]; and (6) willing to participate in any arm of the study.

Exclusion criteria are considered: (1) inability or unwillingness to give informed consent for participation; (2) absence of internet access or internet-enabled device; (3) presence of serious orthopedic, cardiovascular, or cardiopulmonary conditions that limit physical exercise participation; (4) major psychiatric or cognitive disorders; or (5) were otherwise not cleared by their oncologist.

### Interventions

A detailed description of both interventions is provided below:

### Multicomponent training and health education group (MTHE)

The MTHE will be performed twice a week over 12 weeks, on pre-established and non-consecutive days. The sessions will be held by video call on Google Meet platform (approximately 60 min of duration) in small groups (maximum of three patients) and are supervised by physical education students or professionals previously trained to carry out the intervention. The first session of the training program will be performed individually (i.e., 1:1 supervision ratio) to ensure adequate orientation. All equipment and materials will be delivered to participants at the beginning of the intervention (i.e., two pairs of 2- and 4-kg dumbbells and one exercise mattress). The exercises within a training session will be ordered as follows: warm-up (joint mobilization and aerobic stimulus), balance exercise, resistance exercise, and stretching of the main muscles used during the training session. The training periodization used throughout the 12 weeks can be seen in Table 1.

**Table 1:** Training periodization and exercises used throughout the 12 weeks intervention in the MTHE group

Volume	Weeks 1–4	Weeks 5–8	Weeks 9–12
Joint mobilization: 2 sets × 10 repetitions each	Joint mobilization: Stiff and shoulder front raise	Joint mobilization: Stiff and shoulder front raise	Joint mobilization: Stiff and shoulder front raise
Aerobic stimulus: 2–3 sets × 20 s	Aerobic (2 sets): Skipping	Aerobic (2 sets): Adapted Jumping jacks	Aerobic (3 sets): Skipping (session A); Jumping jacks (session B)
Balance exercises: 2 sets × 20 s	Balance: Single leg stance (Right leg + Left leg)	Balance: Imaginary line walking	Balance: Airplane Pose (Right leg + Left leg)
Resistance exercises: 2–3 sets × 10–15 repetitions 10 exercises	Resistance (2 sets × 10–12 repetitions, 5–6 OMNI) exercises (a):	Resistance (2–3* sets × 10–12 repetitions, 6–7 OMNI) exercises (b):	Resistance (2–3* sets × 12–15 repetitions, 7–8 OMNI) exercises:
Static stretching exercises and health education once a week	Dumbbell floor press, Pelvic lift, One-arm dumbbell row, Chair squat, Lateral raises, Split squat Biceps curl, Crunch, Lying triceps extension Isometric crunch	Dumbbell fly*, Unilateral pelvic lift, One arm wide grip dumbbell row*, Dynamic plus isometric squat, Front raise, Lunge*, Hammer curl, Dynamic plus isometric lying triceps extension, Isometric reverse crunch, Bird dog	First session of the week: exercises (a) (Dumbbell floor press*, One arm row*, Split squat*) Second session of the week: exercises (b) (Dumbbell fly*, One arm wide grip dumbbell row*, Lunge*)

In the resistance exercises labeled with “*” 3 sets are performed; in the remaining exercises, 2 sets. In exercises labeled as “dynamic plus isometric” 10 dynamic repetitions plus 5 to 10 s of isometric contraction of the same exercise are performed per set; in exercises labeled as isometric only, each set will last 10 to 15 s

The warm-up is comprised of joint mobilization in low intensity (i.e., using a stick or broomstick as load) and the aerobic stimulus through skipping exercise and adapted jumping jacks (restricting shoulder abduction until 90°, to prevent discomforts related to surgery or treatment). The warm-up will last for approximately 7 min.

In the main part of the session, balance exercises will be performed through single-leg stance, progressing to walking in an imaginary line, and airplane poses in subsequent mesocycles. Thereafter, ten resistance exercises will be performed in pairs alternated by body segment (upper limbs–lower limbs). The intensity will be controlled through an individual’s Rating of Perceived Exertion (RPE), ranging from 5 to 8 on an OMNI scale of 0 to 10 in which zero is no effort (rest), and 10 is a maximal effort [36]. The investigators will use the following question after each exercise: “how hard do you feel your muscles are working?”. Dumbbells will be used for resistance exercises, except for core, pelvic lifting, and split squat exercises. Exercises will change every 4 weeks. The exercises performed in each mesocycle are presented in Additional file 0^1^. The exercises will be performed with a 20:20 cadence (i.e., 2 s of eccentric phase, no pause during the transition, and 2 s of concentric phase). When the intended RPE is not achieved, the cadence will be increased to 40:20, and/or the external load will be increased. The last strategy to achieve the desired RPE is to perform 2 s of isometric contraction on the most challenging angle of the exercise movement in each repetition. The exercises will be alternated by segments, with 30-s rest between exercises and 90-s rest between sets for the same exercise. Due to fluctuations in exercise tolerance, capacity, and self-efficacy during treatment, a flexible prescription allowing patients to auto-regulate the session’s intensity according to their condition in each session will be ensured [22]. Instructional videos of exercises will be sent to participants of the MTHE before the video call session. In the last mesocycle, the exercises are a combination of those performed in the first 8 weeks. The main part of the training session will last from 38 to 50 min.

The cool-down phase will last 5–10 min, during which participants will perform static stretching exercises on major muscle groups (pectoralis major, latissimus dorsi, hamstring, adductor, and glutes) for one set of 20 s each. Additionally, in the second training session of each week, after stretching exercises, the supervisor will address one health education topic, based on the same contents planned for the health education group, but more briefly, lasting 15–20 min. All participants will be instructed to report difficulties and limitations, and training variables (i.e., RPE, load, cadence, and total volume) or any exercise adaptation are registered.

### Health education group (HE)

In the HE, participants receive a folder via WhatsApp or e-mail with information related to breast cancer management once a week. In addition, 2 days after, the ABRACE team and a small group of participants (i.e., maximum of three patients) discuss the target topic of the week in an online session, via videoconference, lasting approximately 30 min. The first session of the health education program will be delivered individually (i.e., 1:1 supervision ratio) to provide a more comfortable environment for the participant to express cancer-related barriers and concerns. The topics approached in the 12 weeks are the following: anxiety and depressive symptoms, fatigue, body image, body composition and bone health, symptoms in the arm and breast, vasomotor symptoms, peripheral neuropathy, pain and arthralgia, sexual dysfunction, quality of life, physical activity, and eating habits. Also, the conversations aim to encourage the participants to increase their physical activity levels following the current guidelines of 150 min per week of moderate aerobic exercise [9]. The MTHE will receive the same folder.

#### Criteria for discontinuing allocated interventions

Participants may be discontinued from the study for safety reasons or withdrawal of participant’s consent. For participants allocated to any group, medical advice, disease complication, or a severe health event during the study, which precludes attendance to intervention sessions, are considered criteria for interrupting participation in the study. Even participants who have discontinued the interventions will continue to be contacted by the study and invited for final evaluations.

#### Strategies for trial retention

Participants allocated to both groups will receive text messages to reinforce the date and time of interventions 2 days before each session. We will use phone calls or WhatsApp messages to ask participants about their reasons for not attending the session. There are no prohibited concomitant interventions; however, any systematic or supervised physical exercise should be reported by the participants and documented for future discussions.

#### Outcomes

Standardized methodological procedures for outcomes assessment are listed below.

### Primary outcome

The primary study outcome is cancer-related fatigue, which will be assessed using the Piper fatigue scale. Cancer-related fatigue was chosen as the primary outcome because it is a widespread side effect caused by chemotherapy, and it is identified among high-priority cancer- and treatment-related toxicities based on its potential to affect cancer care rapidly [28, 38].

### Secondary outcomes

Additional outcomes that are clinically relevant for breast cancer patients will be assessed by online questionnaires, including quality of life, symptoms of depression and anxiety, physical activity level, and cancer-related cognitive function. In addition, a functional performance test will be held by video call. These outcomes are evaluated before and after 12 weeks of intervention.

### Other outcomes

Other outcomes include sociodemographic, clinic characteristics, and self-reported anthropometric measures assessed at baseline. After 12 weeks of intervention, a follow-up questionnaire evaluating the individual motivation perception, lifestyle changes, and main barriers to group participation will be carried out. Also, adherence to interventions will be reported as group attendance and compliance with protocol sessions.

### Safety outcomes

A medical clearance for physical exercise provided by their oncologist is requested for each patient. We will collect and manage spontaneously reported adverse events. Such events will be classified according to their severity (i.e., mild, moderate, severe), predictability (i.e., expected or unexpected), and potential relation with study procedures (i.e., definitely related, possibly related, or unrelated) [37]. All cases of adverse events will be discussed and, if necessary, adjudicated by at least two out of the following investigators: principal investigator (S.P.), study manager (J.H.), and medical and expert consultants.

#### Sample size

The sample size estimation was based on the number of patients feasible to collect given our financial resources during the period of the study (concomitant with the COVID-19 pandemic )[29]. We estimate that the total sample will be around 30 participants. Therefore, approximately 15 women will be enrolled in each group.

#### Assignment of interventions and blinding

After consent, each participant will receive an internal identifier number. After completing the baseline assessments, participants are randomized to either the MTHE or HE. A blinded investigator created a computer-generated randomization sequence, via random function (Excel software), 1:1 ratio, with a block of random sizes that are not disclosed to ensure concealment. The allocation concealment is implemented by researchers in charge of requesting randomization for one of the blind investigators with access to the randomization list (C.B., S.P.), via email, from the identifier number. A researcher (J.H.) allocates individuals in the groups by phone call or WhatsApp message. Randomization requests follow the order in which participants complete the baseline assessments. The allocation date of the participants is recorded in an Excel spreadsheet.

Blinding will be applied for assessors of all primary and secondary outcomes, except physical activity level and cognitive function (the researcher monitoring these two evaluations is not blinded, but both questionnaires are self-administered). Participants are asked to omit their assigned group and not talk about their interventions during the outcome assessments. Due to the nature of the intervention, the study staff supervising training sessions and participants are non-blinding. In unblinding cases, the principal investigator will be notified with the participant ID, date, and reasons for unblinding.

#### Data collection

On the first day, previous physical activity levels and cognitive function will be assessed. After at least a 48-h interval from the first day, primary and all other secondary outcomes will be measured. During the assessments a video call (Google Meet) will be held with each participant individually. The evaluators were trained before starting the trial, and periodic meetings and written communication between the evaluators will be established fortnightly to promote internal transparency and consolidate data collection procedures. The time scheme for study conduction is presented in Table 2.

**Table 2:** Time scheme for ABRACE: Telehealth conduction

Study period							
	Enrolment and baseline measures	Baseline measures	Allocation	Post-allocation		Close out	
**Timepoint**	**t**_**0**_	**t**_***1***_	***t***_***2***_	***t***_***3***_	***t***_***4***_	***t***_***5***_	***t***_***6***_
Timepoint description	Interview and baseline evaluations day 1	Baseline evaluation day 2	-	Intervention start	Intervention end	Final evaluation day 1	Final evaluation day 2
**Enrolment**							
Eligibility screening	x						
Informed consent	x						
Allocation			x				
**Interventions**							
Multicomponent Training Group plus Health Education				x	x		
Health Education Group				x	x		
**Assessments**							
Primary outcomes							
Cancer-related fatigue		x					x
Secondary outcomes							
Quality of life		x					x
Depression symptoms		x					x
Anxiety symptoms		x					x
Functional test		x					x
Cognitive function	x					x	
Physical activity level	x					x	

#### Assessment of primary outcome

##### Cancer-related fatigue

Cancer-related fatigue is determined through an interview by scores from the Piper Fatigue Scale, the Portuguese-validated version [35, 41]. Piper Fatigue Scale consists of 22 items, ranging from 0 (no fatigue) to 10 (severe fatigue), to assess four dimensions of subjective fatigue and total fatigue. Six items will evaluate behavioral fatigue (i.e., impact of fatigue on school or work, interacting with friends, and the overall interference with enjoyable activities). The affective fatigue includes five items that assess the emotional meaning attributed to fatigue. Other five items comprise the sensory fatigue (i.e., mental, physical, and emotional fatigue symptoms). The cognitive/mood fatigue includes six items to assess the impact of fatigue on concentration, memory, and the ability to think clearly. Total fatigue is measured by averaging these four dimensions.

#### Assessments of secondary outcomes

##### Quality of life

The Brazilian version of the self-report 30-item EORTC QLQ-C30 (version 3.0) will be used to assess the quality of life [1, 33]. Scores will be derived and scaled from 0 to 100 according to the EORTC scoring manual [16], considering the global health status scale and five multi-item functional scales (i.e., physical, emotional, role, cognitive, and social function), with higher scores indicating better quality of life, and also, three multi-item and six single-item symptom scales, with higher scores representing higher levels of complications. In addition, the supplementary 23-item breast cancer-specific module (EORTC QLQ-BR23) is also applied and scored as well. The QLQ-BR23 incorporates five multi-item scales to assess body image, sexual functioning, systemic therapy side effects, breast symptoms, and arm symptoms. In addition, single items will assess sexual enjoyment, future perspective, and hair loss. The scoring approach for this questionnaire is identical in principle to that for the function and symptom scales of the QLQ-C30, where a higher score for the functional scales represents a healthier level of functioning, while a higher score for the symptom scales represents a higher level of symptomatology.

##### Depressive symptoms

Depressive symptoms will be assessed by the Center for Epidemiological Studies – Depression (CES-D) scale, the Portuguese-validated version [19, 42]. This self-report questionnaire, previously used in oncology patients [32], comprises 20 items to assess the individual’s current state, asking about the frequency of symptoms in the last 7 days. Each answer is scored according to an order of frequency of symptoms ranging from 0 (rarely or never) to 3 scores (always). The total score ranged from 0 to 60, with higher values indicating a higher level of depression.

##### Anxiety symptoms

Anxiety symptoms are assessed by the State-Trait Anxiety Inventory, a self-report instrument [49]. The questionnaire comprises two parallel scales to evaluate the trait anxiety and state anxiety, each one with 20 items. The scores for individual items ranged from 1 (“almost never”) to 4 (“almost always”), with a total score ranging from 20 to 80 for each scale.

##### Physical activity level

Self-reported physical activity level is assessed using a translated and validated Brazilian version of Godin-Shepard questionnaire [18, 46]. Participants should report the number of times per week that they engage in vigorous, moderate, and light physical activity over a period greater than 15 min. The frequency is multiplied by a specific coefficient for each intensity, which corresponds to the metabolic equivalent of the task (MET). High scores indicate a higher level of physical activity during free time.

#### Cancer-related cognitive function

The FACT-Cognitive Function [56] is a self-reported questionnaire used to assess cognitive complaints in cancer patients. It consists of four blocks of questions that must be interpreted by assigning answers from 0 (“never”) to 4 (“several times a day”) for the last 7 days. The score is presented in subscales referring to perceived cognitive deficits, comments from other people, perceived cognitive skills, and quality of life impact. Higher scores represent a better cognitive function.

##### Functional capacity

The lower limb functional performance is assessed through the sit-to-stand test [43]. The participant starts seated in the chair, with her back supported and her feet shoulder-width apart and fully supported on the floor. The upper limbs will be crossed against the chest. After two test attempts, at the evaluator’s “start” signal, the participants get up and sit down as many times as possible for 30 s, with correct execution. The intra-rater reliability for this test is reported as 0.88 in a previous study [58] evaluating sit and stand test remotely by video in older adults with cancer.

#### Other outcomes

##### Sociodemographic and clinical characteristics

A questionnaire will be applied to assess health parameters and characterize the sample. Sociodemographic factors and data on histological type of tumor, stage, the status of hormone receptors, expression of HER-2 and chemotherapy, immunotherapy, or radiation therapy protocols used will be collected and/or confirmed directly with the responsible doctor, with prior authorization from each patient.

##### Anthropometric measures

The baseline anthropometric measures (i.e., height and body mass) will be self-reported by the participants due to the impossibility of virtual assessment.

##### Follow-up questionnaire

An adaptation in the questionnaire used in Schott et al. [47] was made to evaluate important outcomes in cancer patients such as safety, fun, motivation, future, benefits for daily life, intervention partner influence, training-related exhaustion, satisfaction, self-confidence on physical performance, supervision preference, changes in lifestyle including physical activity and eating habits, and main barriers to group participation. Participants will answer 14 questions about the individual perception of the intervention using a 7-point Likert scale from 1 (i.e., do not agree at all) to 7 (i.e., I entirely agree). The follow-up questionnaire is available in Additional file 0^2^.

##### Adherence assessments

Adherence is recorded as group attendance and compliance in both interventions. Adherence will be treated by absolute and relative frequency of the number of sessions performed. Compliance will be treated as the number of sessions in which the full protocol was achieved.

#### Data management

Questionnaires data will be collected by online forms (Google Forms), and the functional performance test scores will be filed on a Google Sheet. All data will be stored in the Google Drive linked to a specific email for the trial that only researchers involved have access to. Data entry is carried out for two investigators individually (J.H. and S.P.) and will remain in their ownership. Audition for missing or inaccurate data will be conducted when necessary.

#### Statistical considerations

Generalized Estimating Equations (GEE) and Bonferroni post hoc tests will be used for comparison between time points (baseline and post-intervention) and groups (MTHE and HE) for both per-protocol and intention-to-treat analyses. The per-protocol analysis (PP) will count all women with training frequency ≥ 70% of intervention sessions (≥ 16 sessions for participants allocated in the exercise program, and ≥ 8 sessions for participants allocated to the education program). For the intention-to-treat analyses, the incomplete data (i.e., missing values) will be estimated using maximum likelihood estimation by the automatic regression imputation method.

Additionally, we plan to carry out subgroup analysis stratifying both groups by clinical characteristics of the tumor (histological type of tumor, tumor staging, and status of hormone receptors) and treatment (chemotherapy and radiotherapy protocols). Considering that withdrawals may occur due to specific harms or other non-anticipated reasons, we will assess whether additional grouping (indicator) should be made due to different missing data patterns. Therefore, we will qualitatively document reasons and details of withdrawals on a case-by-case basis. Continuous variables will be summarized according to intervention groups at baseline, if applicable, and end of trial using arithmetic or geometric means, standard deviations, ranges, and interquartile ranges as appropriate. According to intervention groups, categorical variables at baseline and end of the trial (if applicable) will be summarized as absolute number and proportion of subjects (%). Standardized effect sizes will be calculated by dividing the between-group difference of the post-intervention means by the pooled baseline standard deviation. The significance level established for all analyses will be *p* < 0.05, and all statistical procedures will be analyzed and processed using the SPSS version 22.0 software.

#### Monitoring

##### Data monitoring and auditing

The ABRACE: Telehealth Study does not have a data monitoring committee either planned auditing trial conduct due to limited resources. We reason that this committee would not be mandatory due to the characteristics of interventions and outcomes, despite its high value for the overall quality of the trial. J.H., R.P., and S.P. will have access to all data and interim analysis and make the final decision to terminate the trial.

##### Harms, ancillary, and post-trial care

The adverse events will be managed according to the National Institute of Aging (40). A serious adverse event is any unfavorable medical occurrence, with risk of death or disability of the participant, which requires hospitalization. The identification, possible solutions, and documentation of adverse events are based on discussion and analysis between the principal investigator, study directors, study managers, and medical team. For any injuries suffered during trial enrollment related to the study (after adjudication by these researchers), we plan contingency actions to provide participants primary health care and guidance. Every effort will be made to prevent any unwanted events.

##### Dissemination policy

We aim to disseminate the methods and findings of the ABRACE: Telehealth Study to as many stakeholders as possible. Therefore, our dissemination plan after trial completion includes inviting the participants to watch presentations about study design, findings, and interpretation, and the results of this study, whether positive, negative, or inconclusive, will be submitted to a peer-reviewed international journal for possible publication. All information regarding the full protocol, participant-level dataset, and statistical code will be available from the corresponding author on reasonable request.

## Discussion

This trial is strengthened in the attempt to propose an exercise protocol performed at home, via telehealth, and test its effectiveness in relevant outcomes for the quality of life of patients with breast cancer. The importance of exercise during and after treatment of these patients is becoming increasingly apparent, and it is essential that professionals in the field of physical exercise find strategies to reduce the barriers to practice and increase adherence. Home-based programs that incorporated technology to support interventions reported attendance rates greater than 70% [34]. In addition, this study is enhanced by using guided exercise with supervision. Home-based exercise does not necessarily mean “unsupervised” exercise [31]. Currently, videoconferencing platforms allow exercise professionals to demonstrate and provide exercise guidance virtually in individual or group sessions [5].

The feasibility and quality of telehealth exercise interventions have been underexplored, and their effectiveness remains unclear in the exercise oncology setting [5, 44]. Previous studies using technology to apply interventions with physical activity in the cancer population have reported improvements in outcomes such as physical activity levels, weight loss, and high adherence [20, 34, 44]. Notwithstanding, the effectiveness in outcomes related to fatigue, quality of life, anxiety, and depressive symptoms remain unidentified [34, 44]. The only study that investigated exercise intervention via telehealth using a face-to-face videoconference platform to supervise cancer survivors directly reported 94% adherence in those who choose to participate in the training group [13]. However, the authors did not find improvements in sedentary time or physical activity in the participants (during COVID-19 lockdown), nor evaluated outcomes directly related to side effects of treatment.

Telehealth interventions may optimize cancer care due to the potential to enhance adherence rates, remove barriers, support access and motivation, reduce costs, be time-efficient, and combine both clinician and participant feedback [5, 34]. Ongoing trials have contributed to exploring the positive effects of online exercise programs in the oncology population. Currently, a randomized controlled trial employing jumping training delivered by recorded videos and including behavioral and diet counseling has been implemented to improve bone health and clinical outcomes in pediatric cancer survivors [17]. Furthermore, an innovative single-arm study assesses the feasibility and acceptability of a group-based exercise intervention using teleconferencing in older patients who have received or are undergoing liver cancer treatment [40]. However, web-based interventions targeting behavior change through real-time monitoring and assisting exercise techniques, progressions, and compliance to mitigate cancer-related fatigue and promote a better quality of life and mental health still need more investigations. To our knowledge, this is the first randomized clinical trial using face-to-face videoconference to supervise exercise evaluating clinically relevant outcomes directly related to cancer treatment in oncology participants.

## Trial status

The recruitment period for the ABRACE: Telehealth study is planned to range from March 2021 to March 2023. This is the first version of the manuscript and is accompanied by a description of existing amendments (Additional file 0^3^).

## Supplementary Information

**Additional file 1.** Exercises in mesocycles I, II and III.

**Additional file 2.** follow-up questionnaire.

**Additional file 3.** Amendment’s chronology.

**Additional file 4.** Spirit Checklist.

**Additional file 5.** Roles of InvestigatorsR2.

## Data Availability

Not applicable. This manuscript does not contain any data.

## Abbreviations

ABRACE Telehealth: Adaptations to Breast Cancer and Exercise Using Telehealth
MTHE: Multicomponent Training Group plus Health Education
HE: Health education group
RPE: Rating of perceived exertion
