# Supplementary material for: Telehealth multicomponent exercise and health education in breast cancer patients undergoing primary treatment: rationale and methodological protocol for a randomized clinical trial (ABRACE: Telehealth)
Source: Trials. 2023 Jan 19;24:42. doi: 10.1186/s13063-022-07015-z (PMC9851110; doi:10.1186/s13063-022-07015-z)
Supplement: Supplementary file 1 — Additional file 1. Exercises in mesocycles I, II and III. [file 13063_2022_7015_MOESM1_ESM.pdf]

BOARD - EXERCISES

**MESOCYCLE 1**		**MESOCYCLE 2**	
PHASE 1	PHASE 2	PHASE 1	PHASE 2
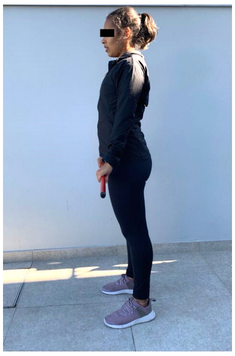	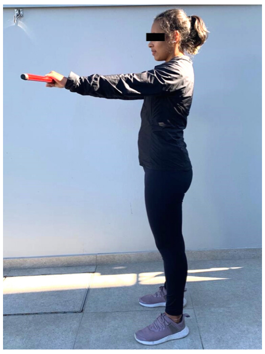	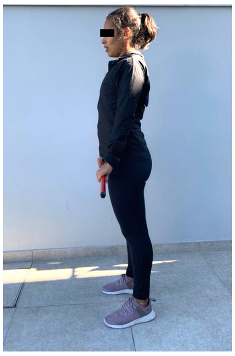	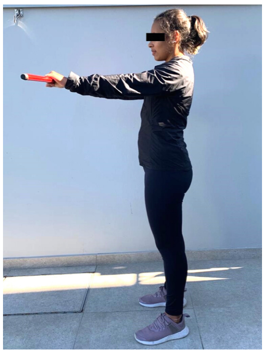
Shoulder front raise		Shoulder front raise	
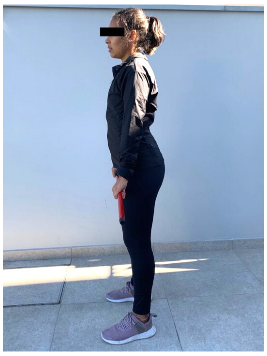	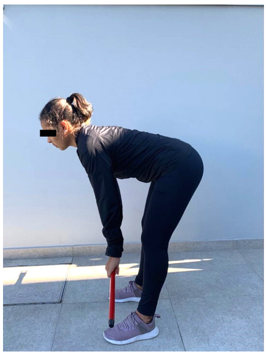	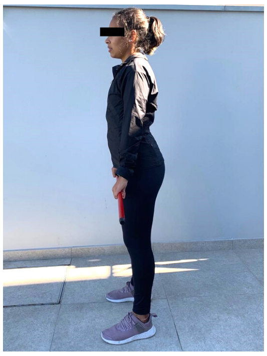	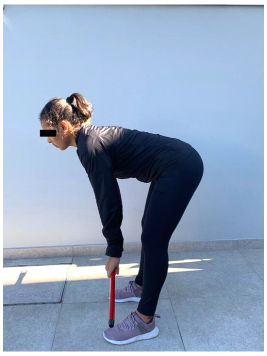
Stiff		Stiff	
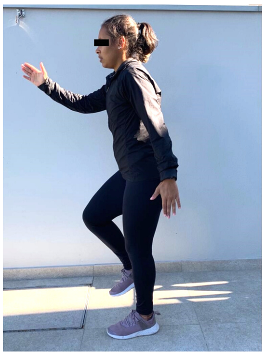	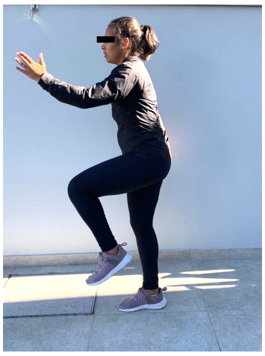	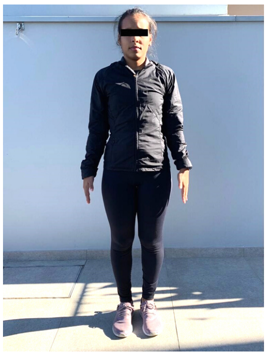	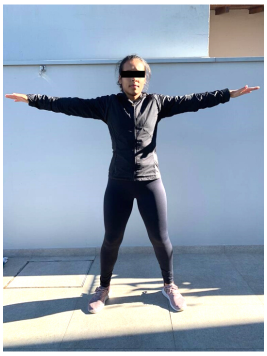
Skipping		Adapted jumping jacks	
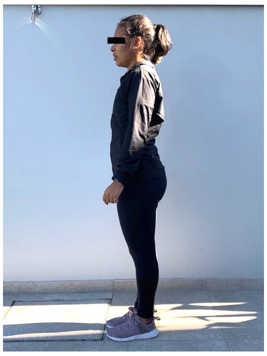	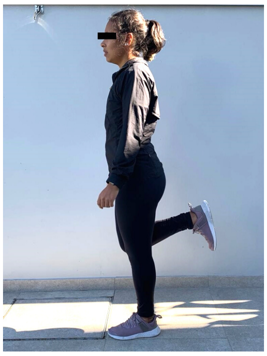	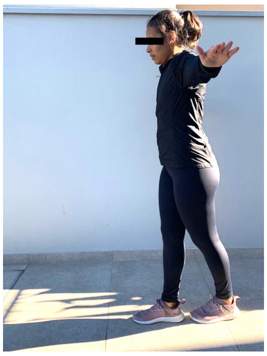	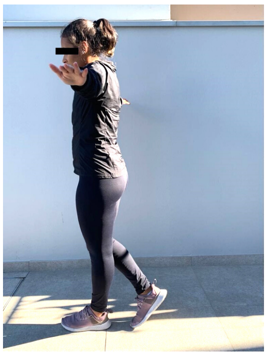
Single leg stance (Right leg + Left leg)		Imaginary line walking	

BOARD - EXERCISES

MESOCYCLE 1		MESOCYCLE 2	
PHASE 1	PHASE 2	PHASE 1	PHASE 2
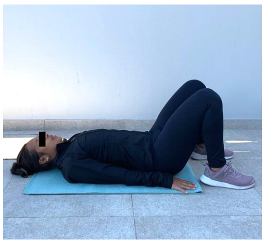	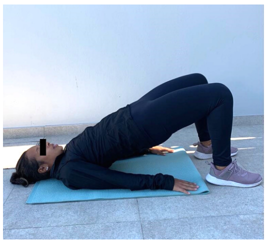	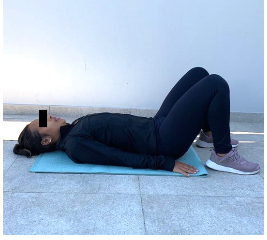	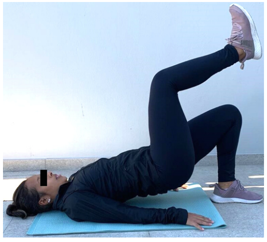
Pelvic lift		Unilateral pelvic lift	
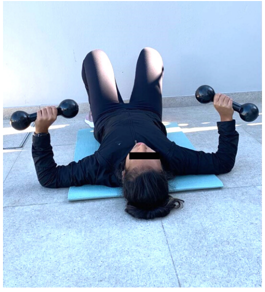	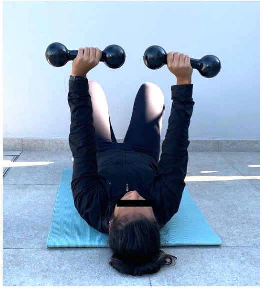	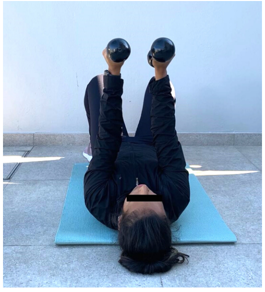	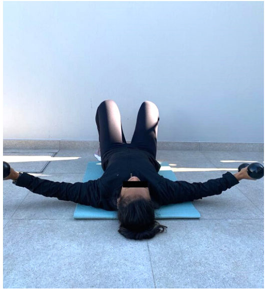
Dumbbell floor press		Dumbbell fly	
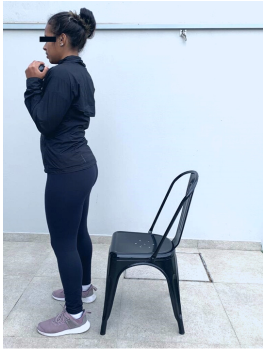	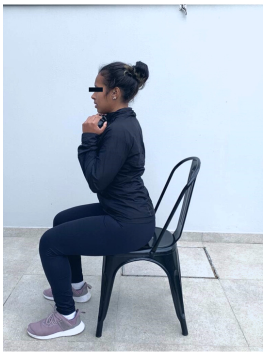	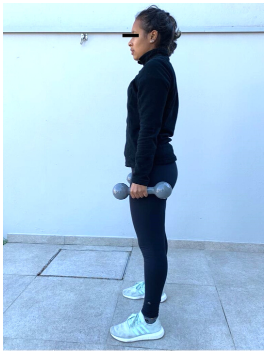	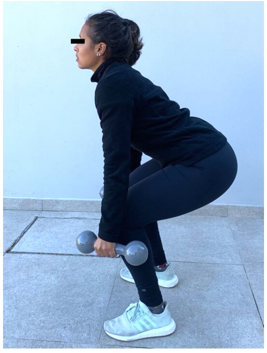
Chair squat		Dynamic plus isometric squat	
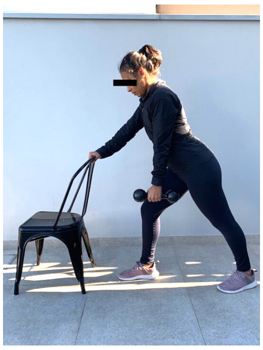	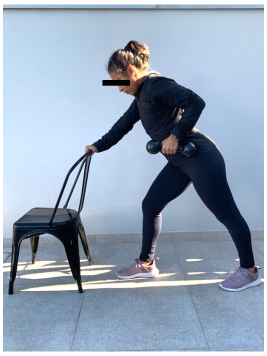	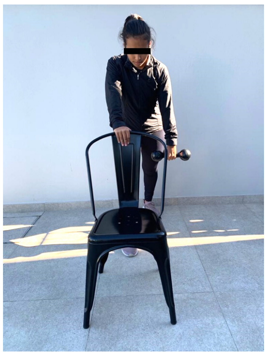	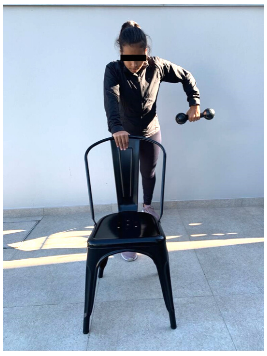
One-arm dumbbell row		One arm wide grip dumbbell row	

BOARD - EXERCISES

**MESOCYCLE 1**		**MESOCYCLE 2**	
PHASE 1	PHASE 2	PHASE 1	PHASE 2
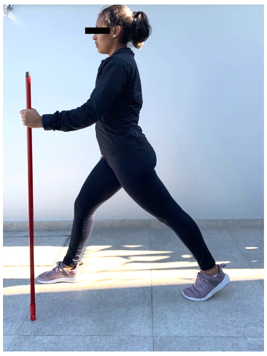	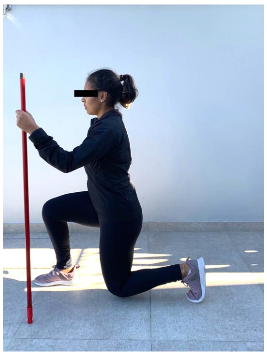	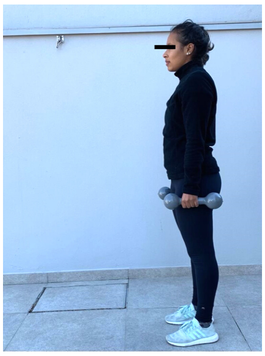	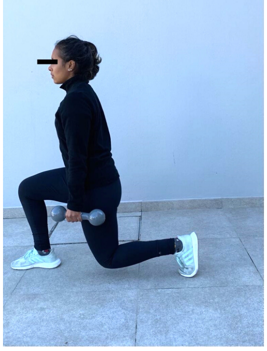
Split squat (w/ stick support to balance)		Lunge	
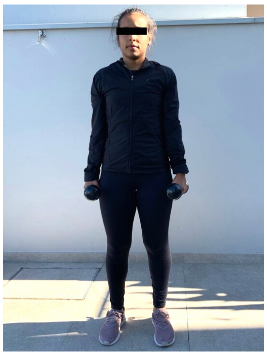	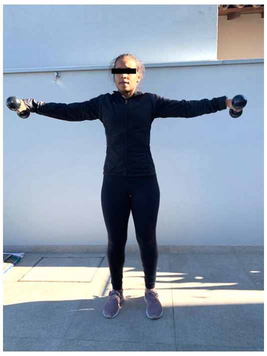	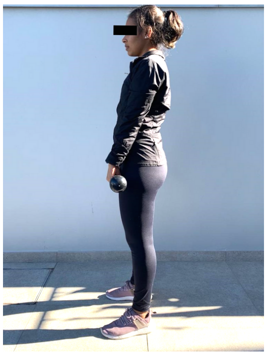	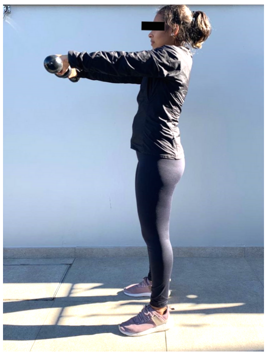
Lateral raises		Front raise	
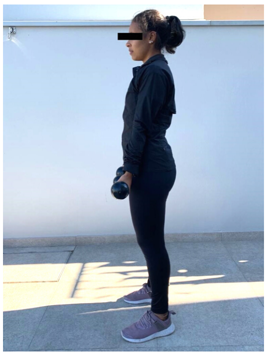	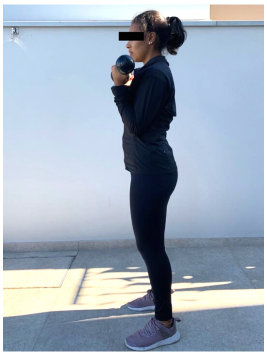	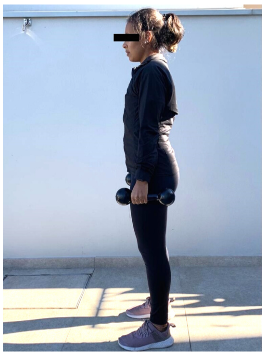	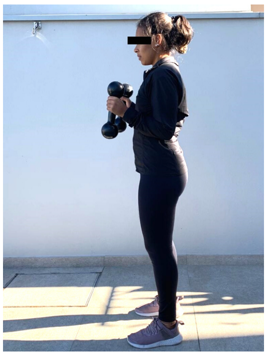
Biceps curl		Hammer curl	
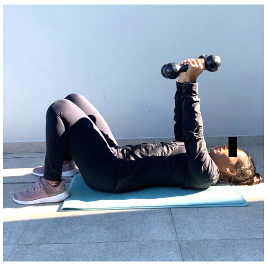	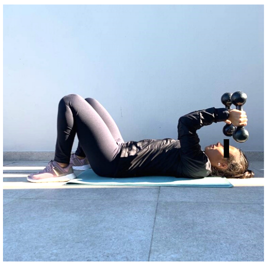	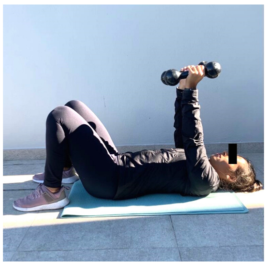	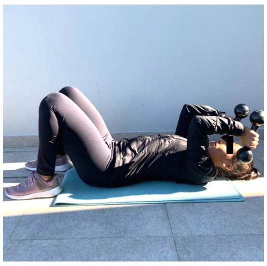
Lying triceps extension		Dynamic plus isometric lying triceps extension	

BOARD - EXERCISES

<table>
<tr><th colspan="2">MESOCYCLE 1</th><th colspan="2">MESOCYCLE 2</th></tr>
<tr><td>PHASE 1</td><td>PHASE 2</td><td>PHASE 1</td><td>PHASE 2</td></tr>
<tr><td colspan="2">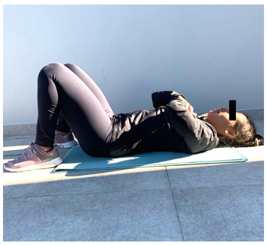 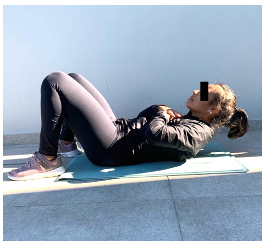
Crunch</td><td colspan="2">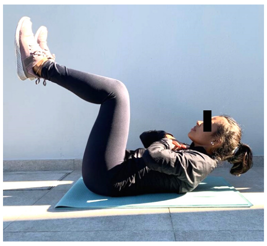
Isometric reverse crunch</td></tr>
<tr><td colspan="2">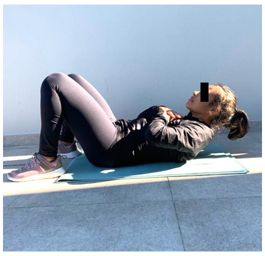
Isometric crunch</td><td colspan="2">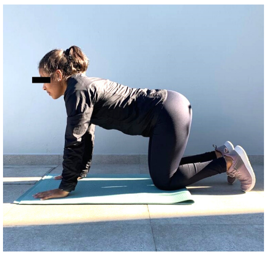 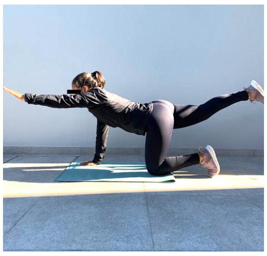
Bird dog</td></tr>
</table>

<table>
<tr><th colspan="2">MESOCYCLE 3</th></tr>
<tr><td>PHASE 1</td><td>PHASE 2</td></tr>
<tr><td colspan="2">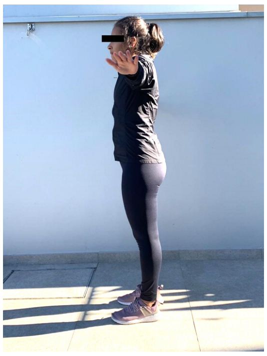 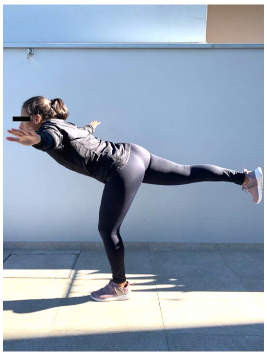
Airplane pose (Right leg + Left leg)</td></tr>
</table>
