# Supplementary material for: Telehealth multicomponent exercise and health education in breast cancer patients undergoing primary treatment: rationale and methodological protocol for a randomized clinical trial (ABRACE: Telehealth)
Source: Trials. 2023 Jan 19;24:42. doi: 10.1186/s13063-022-07015-z (PMC9851110; doi:10.1186/s13063-022-07015-z)
Supplement: Supplementary file 2 — Additional file 2. follow-up questionnaire. [file 13063_2022_7015_MOESM2_ESM.docx]

**Additional file 02**

**Follow-up questionnaire**

You will be asked to answer 14 questions about the participants' perception of the intervention and essential outcomes in primary cancer treatment. A 7-point Likert scale will be used with 1(=do not agree at all) to 7 (=I entirely agree).

**1. My participation in the intervention group (training or health education) influenced me to a behavior change.**

Do not agree at all I entirely agree

( ) ( ) ( ) ( ) ( ) ( ) ( )

1 2 3 4 5 6 7

**2. My participation in the intervention group influenced me to achieve higher levels of physical activity.**

Do not agree at all I entirely agree

( ) ( ) ( ) ( ) ( ) ( ) ( )

1 2 3 4 5 6 7

**2.1 In general, were changed habits or behaviors?** _______________________________________________________________________

**3. My participation in the intervention group influenced me to have better eating habits.**

Do not agree at all I entirely agree

( ) ( ) ( ) ( ) ( ) ( ) ( )

1 2 3 4 5 6 7

**4. I feel that I participated in the study.**

Do not agree at all I entirely agree

( ) ( ) ( ) ( ) ( ) ( ) ( )

1 2 3 4 5 6 7

**5. I feel safe performing physical activity.**

Do not agree at all I entirely agree

( ) ( ) ( ) ( ) ( ) ( ) ( )

1 2 3 4 5 6 7

**6. During the intervention sessions, I felt better or more motivated with a partner together (another patient as a colleague).**

Do not agree at all I entirely agree

( ) ( ) ( ) ( ) ( ) ( ) ( )

1 2 3 4 5 6 7

**7. The intervention was fun.**

Do not agree at all I entirely agree

( ) ( ) ( ) ( ) ( ) ( ) ( )

1 2 3 4 5 6 7

**8. My physical activity motivation has increased significantly through interventions.**

Do not agree at all I entirely agree

( ) ( ) ( ) ( ) ( ) ( ) ( )

1 2 3 4 5 6 7

**9. I find physical activity very strenuous.**

Do not agree at all I entirely agree

( ) ( ) ( ) ( ) ( ) ( ) ( )

1 2 3 4 5 6 7

**10. I will start/continue with strength training in the near future.**

Do not agree at all I entirely agree

( ) ( ) ( ) ( ) ( ) ( ) ( )

1 2 3 4 5 6 7

**11. Physical activity makes everyday life easier for me.**

Do not agree at all I entirely agree

( ) ( ) ( ) ( ) ( ) ( ) ( )

1 2 3 4 5 6 7

**12. Proper care and a good coach are very important for me in physical exercise.**

Do not agree at all I entirely agree

( ) ( ) ( ) ( ) ( ) ( ) ( )

1 2 3 4 5 6 7

**13. Physical activity gives me increased self-confidence in my physical performance.**

Do not agree at all I entirely agree

( ) ( ) ( ) ( ) ( ) ( ) ( )

1 2 3 4 5 6 7

**14. Which were the main barriers to great participation in your intervention group? Please, select 0 (zero) to 3 (three) options.**

( ) Treatment adverse effects

( ) Lack of motivation

( ) Lack of time

( ) Inaccessibility of technology (computer, mobile, or internet)

( ) Lack of familiar and friends support to my participation

( ) unexpected consults and exams

( ) unforeseen or constantly changes in my work schedule

( ) Other

**14.1 If applicable, specify which medication side effect(s) made it more difficult for you to participate:** _______________________________________________________________________
