# Supplementary material for: Telehealth multicomponent exercise and health education in breast cancer patients undergoing primary treatment: rationale and methodological protocol for a randomized clinical trial (ABRACE: Telehealth)
Source: Trials. 2023 Jan 19;24:42. doi: 10.1186/s13063-022-07015-z (PMC9851110; doi:10.1186/s13063-022-07015-z)
Supplement: Supplementary file 3 — Additional file 3. Amendment’s chronology. [file 13063_2022_7015_MOESM3_ESM.docx]

**Additional file 03**

**Amendments chronology**

Protocol amendment number: 01

Authors: Stephanie S. Pinto

Issue date: 18 March 2021

Primary reasons for amendment: modify the eligibility criteria to be in a maximum of 50% of some primary treatment (chemotherapy or radiotherapy), to be in treatment either in chemotherapy, radiotherapy, or immunotherapy.

Additional corrections: We will not evaluate any outcome in the middle of the intervention (week 6). The initial (week 0) and post-intervention (week 13) assessments remain without alterations.

Protocol amendment number: 02

Authors: Stephanie S. Pinto

Issue date: July 2021

Primary reasons for amendment: the follow-up questionnaire will be added to the first day of post-intervention evaluations. This assessment aims to analyze the individual’s perception of the intervention regarding outcomes such as safety, fun, motivation, future, benefits for daily life, intervention partner influence, training-related exhaustion, satisfaction, self-confidence on physical performance, supervision preference, changes in lifestyle including physical activity, eating habits, and main barriers to group participation.

Additional corrections: The measures of adherence to intervention will be considered among the study outcomes, not only as data to control frequency. Also, the evaluations order will be changed due to logistic issues. After the interview, on the first day of evaluations will be assessed physical activity levels, cognitive function, sociodemographic and clinic characteristics (including self-reported anthropometric measures). After at least 48h interval from the first day, primary and all other secondary outcomes are measured. The primary study outcome is cancer-related fatigue, the others secondary outcomes are quality of life, symptoms of depression and anxiety, and functional capacity. In addition, the randomization will not be stratified by the city of the participants and the disease stage.
