# Supplementary material for: Telehealth multicomponent exercise and health education in breast cancer patients undergoing primary treatment: rationale and methodological protocol for a randomized clinical trial (ABRACE: Telehealth)
Source: Trials. 2023 Jan 19;24:42. doi: 10.1186/s13063-022-07015-z (PMC9851110; doi:10.1186/s13063-022-07015-z)
Supplement: Supplementary file 5 — Additional file 5. Roles of InvestigatorsR2. [file 13063_2022_7015_MOESM5_ESM.docx]

**Additional file 5**

**Roles of investigators**

*Coordinator committee*

João S. Henkin, Stephanie S. Pinto, Ronei S. Pinto, Cíntia E. Botton, Gabriel S. Trajano

*Recruitment committee*

João S. Henkin, Stephanie S. Pinto

*Assessment committee*

João S. Henkin, Ricardo S. Gehrke, Gabriella B. Freitas.

*Intervention committee*

João S. Henkin, Mariana S. Simon, Guilherme G. Rocha, Caroline B. Silveira.

*Expert consultants*

Oncology: Alessandra Morelle, MD.
